# Real world outcomes in alveolar soft part sarcomas: experience with an ultra-rare sarcoma from a tertiary care centre in North India

**DOI:** 10.3332/ecancer.2024.1813

**Published:** 2024-12-06

**Authors:** Sanal Fernandes, Sameer Rastogi, Kanu Priya Bhatia, Sindhura Chitikela, Shamim A Shamim, Shivanand Gammanagatti, Adarsh Barwad

**Affiliations:** 1 Department of Medical Oncology, AIIMS, New Delhi 110029, India; 2 Department of Nuclear Medicine, AIIMS, New Delhi 110029, India; 3 Department of Radiodiagnosis, AIIMS, New Delhi 110029, India; 4 Department of Pathology, AIIMS, New Delhi 110029, India

**Keywords:** ASPS, vascular, sunitinib, brain metastasis, atezolizumab

## Abstract

**Background:**

Alveolar soft part sarcoma (ASPS) is a rare, indolent soft tissue sarcoma, with a high predilection for systemic dissemination. This study aims to elucidate the patterns of clinical presentation of ASPS and their treatment outcomes, with emphasis on the use of newer therapeutic agents and their efficacy in advanced ASPS.

**Methods:**

This was a retrospective cohort that included patients with ASPS treated at our institute between 2016 and 2023. Clinicopathological data were obtained from case records and analysed to assess outcomes.

**Results:**

The study included 34 patients (19 males, 15 females) with a median age of 28 (3–72) years. 7 patients presented with localised disease, and 27 with metastatic disease. The most common site of primary was the extremities (73%), and the most common sites of metastasis included the lungs (82%) and bones (21%). Brain metastasis was seen in 7 patients at baseline (25.9%). 90% of patients with metastatic disease received a tyrosine kinase inhibitor in the first-line setting with a median progression-free survival of 12 months. The median overall survival in this subset was 36 months. 7 patients with the advanced disease received immune-checkpoint inhibitors (ICIs) (3-atezolizumab, 4-nivolumab); 2 patients on atezolizumab and 1 on nivolumab continue to be progression free at 20,15 and 52 months, respectively. Patients with brain metastasis were seen to have markedly poor outcomes.

**Conclusion:**

The use of anti-angiogenic agents and ICIs has significantly improved survival in patients with advanced ASPS. Brain metastasis confers poor survival in these patients despite the use of newer agents. This study represents the largest cohort of patients with advanced ASPS from this region.

## Introduction

Alveolar soft part sarcoma (ASPS) is an ultra-rare soft tissue sarcoma (STS) that accounts for less than 1% of all STS. The entity was first described by Christopherson in the early 1950’s as a distinct soft tissue neoplasm found in the extremities in young females, characterised by an alveolar morphology with crystalloid intracellular inclusions. The disease commonly affects patients in the 2nd and 3rd decades of their lives, with a preponderance for the female gender. The most commonly involved sites include the extremities (60%), followed by the trunk (20%) and the head and neck region (10%). The incidence of head and neck ASPS has a slightly higher preponderance in the pediatric population (15%–20%) [1, 2].

Pathologically, the tumour demonstrates nests of round, epithelioid cells with an alveolar arrangement, with the cells showing abundant granular cytoplasm with characteristic periodic acid-schiff-positive crystalline inclusions [3]. The molecular signature of this rare neoplasm is the unbalanced t(x;17) translocation that results in the fusion of the TFE3-ASPL genes that drives tumorigenesis via increased MET signalling. The tumour stains for TFE3 on immunohistochemistry; however, the same is not specific to ASPS.

Clinically, the disease presents as a painless, indolent mass in the lower extremities; however, upto 50% of patients have distant metastasis at presentation with the lung being the most common site of distant disease (90%). The incidence of brain metastasis is higher in ASPS as compared to other STS, and its incidence at presentation can vary from 11% to 19% [4]. Given its indolent nature, patients with localised disease have excellent long-term outcomes with 5-year overall survival (OS) ranging from 80% to 90%. Outcomes associated with advanced disease have traditionally been poor (5 years OS <20%), largely attributable to the relative chemoresistance of these tumours overall response rate (ORR <10%) [5]. Targeted agents (multi-target tyrosine kinase inhibitors (TKIs)) have significantly improved outcomes in advanced ASPS, with pazopanib and sunitinib showing definitive activity in multiple retrospective and phase 2 studies [6, 7]. The TKI anlotinib has been approved as a first-line agent in ASPS in China based on a phase 2 trial that showed an ORR of 46% and a progression-free survival (PFS) of 18 months [8]. The use of immunotherapy has shown encouraging results in ASPS. A phase 2 study of atezolizumab in advanced ASPS showed an ORR of 24% with responders demonstrating a sustained and robust response (42% showed a duration of response >12 months) [9]. The combination of nivolumab plus sunitinib has also shown activity in this setting with a 6-month progression-free rate of 48% [10].

Despite advances in the diagnostic and therapeutic modalities of ASPS, this ultra-rare sarcoma remains underdiagnosed and under-reported in India. Hence, we present data from our centre to delineate the clinical features and outcomes of this disease.

## Methods

This was a single-centre, retrospective study that included patients (pediatric and adult) with pathologically confirmed ASPS who were treated at our centre between the years 2016–2023. If patients were diagnosed at an alternative centre, they were included after a pathological review of tissue blocks at our centre. The diagnosis of ASPS was made on the basis of morphological picture and immunohistochemical staining and was confirmed by two sarcoma pathologists at our institute. Clinical data pertaining to demographic features, sites of disease, radiological presentations and survival outcomes were obtained from case records. For patients with localised disease, data on the feasibility of surgical resection, surgical procedure and margin details and adjuvant therapy, if any, were obtained. In patients with metastatic disease, data on sites of disease, use of systemic therapy (cytotoxic, targeted or immunotherapy) and their associated toxicities and responses was collected. Treatment plans for all cases were discussed at the institute’s multi-disciplinary sarcoma meeting prior to the commencement of therapy. All responses were measured using RECIST criteria with contrast CT/PET-CT as the imaging modality.

Data was expressed using standard descriptive statistics. Means and frequencies were compared using unpaired *T*-test and chi-square tests, respectively. Survival was analysed using Kaplan-Meier curves. PFS was defined as the date of start of therapy to the date of progression/last follow up. OS was defined as the time from the date of registration to the time of death. Statistics were computed using SPSS v.20.

## Results

From January 2016 to December 2022, a total of 34 patients were included in the study with 19 males and 15 females with a median age of 28 years (3–72). The most common site of primary was the extremities (73%) followed by the trunk and viscera (17%) and the head and neck region (8%). In tumours originating from the extremities, there was a preponderance for the lower limbs (82%), with the thigh region being the most common extremity site for primaries (50%). 27 patients (79%) had metastatic disease at presentation with the most common site for metastasis being lung (85%), followed by bones (18%) and the liver (11%). Baseline brain imaging was present in 21 patients, and 7 patients had brain metastasis at presentation (26%) (Table 1).

### Localised disease

All 7 patients with localised disease at presentation underwent wide local excision of the primary (4-R0, 3-R2). The 3 patients who underwent an R2 resection belonged to the pediatric age group (ages 3, 3 and 17 years, respectively). Two of these patients received adjuvant radiotherapy (RT) and subsequently attained a complete response. The third patient, who had an oropharyngeal primary, was started on sunitinib post an R2 resection; he achieved a complete response at 6 months, and continues to be disease free on sunitinib. No recurrences have been reported in the subset with localised disease at a median follow-up of 29 months.

### Metastatic disease

**Figure 1.**
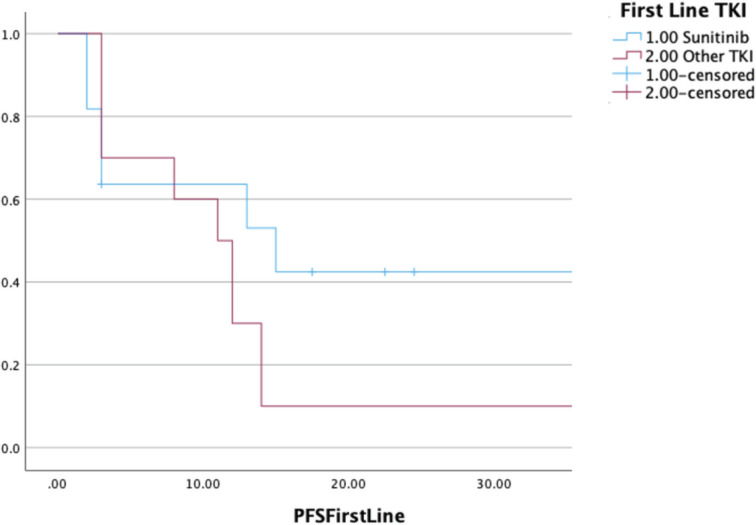
Comparison of PFS in patients receiving sunitinib versus non-sunitinib TKI in the first-line setting (*n* = 24).

Twenty-four patients received TKI monotherapy in the first line setting 14 and 10 patients received sunitinib and a non-sunitinib TKI, respectively. The non-sunitinib TKIs were grouped together for analysis purposes. Non-sunitinib TKIs included pazopanib, sorafenib and crizotinib. The dosing for sunitinib was a daily, continuous 37.5 mg once per day. The ORR and disease control rate (DCR) with sunitinib in the first line setting were 36% and 64%, respectively. The median PFS with TKI monotherapy in the first-line setting was seen to be 12 (9–15) months. Comparison of survival curves between sunitinib and a non-sunitinib TKI in the first line setting in metastatic disease showed numerically better outcomes with sunitinib (median PFS - 15 versus 11 months); however, a statistical significance could not be demonstrated (*p* = 0.14) (Figure 1).

Six patients received sunitinib in the second line setting; the median PFS in this subset was 6 (3–9) months.

The most common adverse effects (AEs) seen with TKI monotherapy included hand-foot skin reaction (29.4%), diarrhoea (23.5%) and hepatitis (11.7%). A majority of these AEs were grade 2 or lesser, and resolved with supportive care and/or temporary withholding of the drug. One patient each developed grade 2 hypothyroidism and grade 3 systemic hypertension requiring medical intervention. Toxicity necessitating dose reduction was not seen in any of the patients.

### Exposure to immunotherapy

**Figure 2.**
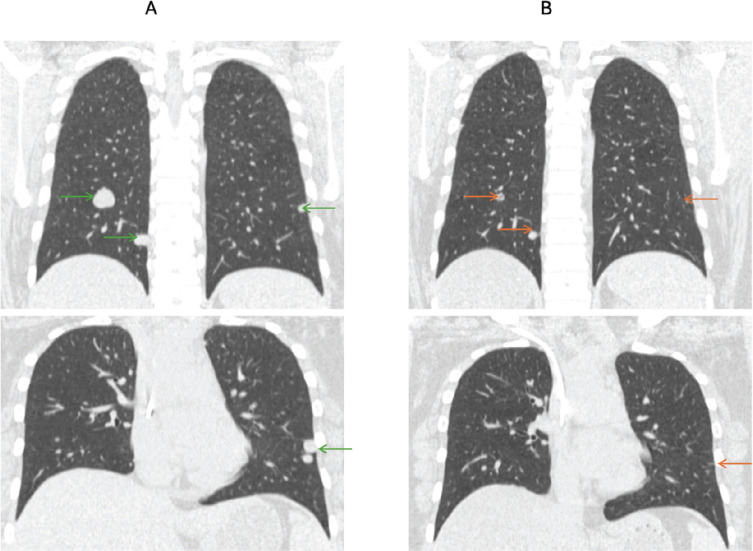
(a): Pre-treatment computed tomography images of a patient with metastatic ASPS with lung metastasis (green arrows). (b): Computed tomography images after 12 months of atezolizumab show significant reduction in previously seen lung nodules (red arrows).

Seven patients with advanced disease received immunotherapy—three patients received atezolizumab in the first-line setting and four received nivolumab in the third or subsequent lines. The best response seen with atezolizumab was a partial response, which was attained after 12 months of therapy (Figure 2). Two patients continued to be progression-free at 20 and 15 months, respectively, while the third patient progressed after a therapy duration of 5 months. Nivolumab was administered in the third or further line setting in four patients. The best response seen with nivolumab was a complete response, which was recorded after 24 months of therapy. The duration of response with nivolumab in the four patients was 3, 5, 5 and 52 months, respectively**.** One patient developed grade 2 hypothyroidism on nivolumab necessitating thyroxine supplementation.

### Survival in patients with baseline brain metastasis

**Figure 3.**
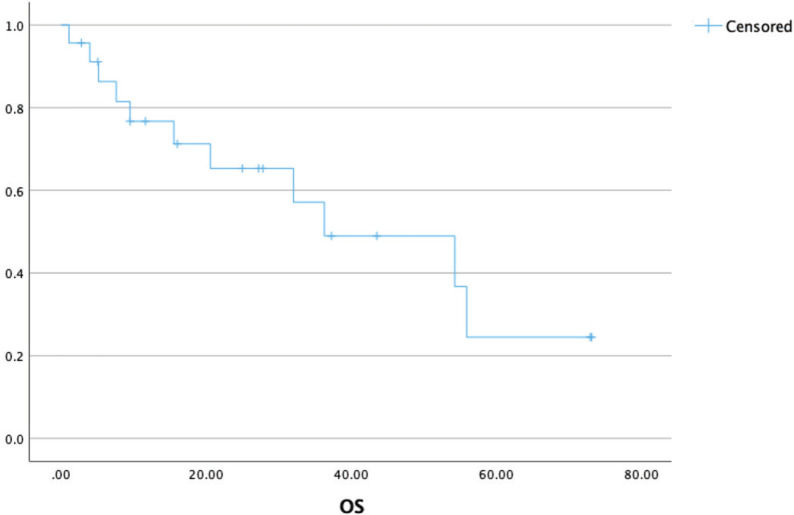
OS in patients with metastatic ASPS (*n* = 27).

**Figure 4.**
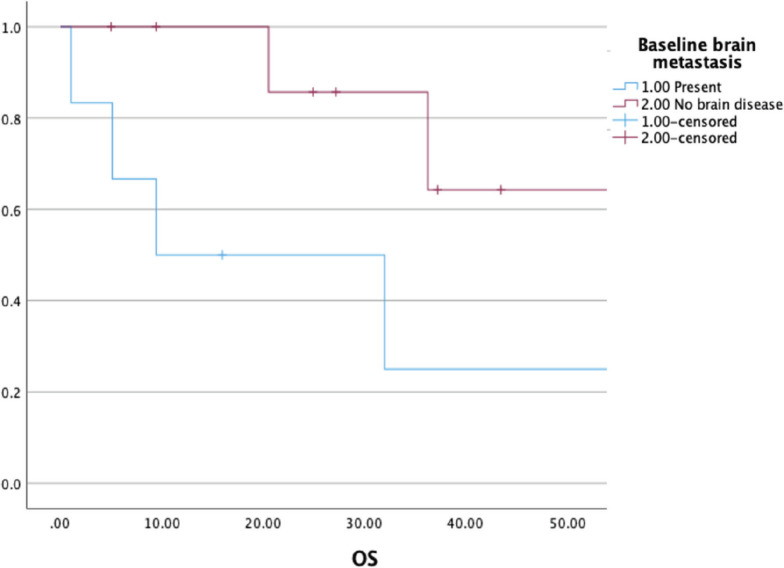
Comparing OS among patients with and without brain metastasis (*n* = 21).

At a median follow-up of 37 (21–54) months, the median OS was 36 (9–54) months with a 3-year OS of 52% (Figure 3). The presence of brain metastasis was seen to confer a significantly poorer survival in the overall population of patients with metastatic disease (median OS of 9.4 versus 56 months, *p* = 0.003) (Figure 4). The outcomes in patients with brain metastasis did not differ with respect to the TKI exposure (sunitinib versus non-sunitinib). One patient with brain metastasis was exposed to immunotherapy (nivolumab); the best response seen was stable disease, with systemic progression at 5 months.

## Discussion

ASPS is a vascular, indolent soft tissue neoplasm, albeit with a high metastatic potential. This is the first study from the region, to the best of our knowledge, to describe the clinical outcomes of ASPS and their response patterns to TKI therapy/immunotherapy. The median age of the patients in our study was 28 years which is in concordance with existing literature. Most studies have shown a female preponderance of the disease; however, our study showed a slightly higher incidence in males [1, 11, 12]. Distal metastasis was seen in 79% of patients at presentation. The higher rate of metastasis described in this study is probably attributable to delayed presentations and the lack of dedicated sarcoma clinics in the country, which contributes to delays in diagnosis and initiation of treatment. ASPS has a higher propensity for brain metastasis as compared to other STS. Our study found the prevalence of brain metastasis to be 26%, which is higher than that in the reported literature [4]. However, brain imaging was available for only 21 of the 27 patients with disseminated disease, and hence this incidence of brain metastasis might not be representative of the actual incidence.

The 5-year survival in advanced ASPS has significantly improved from <30% in the pre-TKI era to 50%–60% in the TKI era [1, 12]. The TFE3-ASPL gene fusion has been shown to upregulate pro-inflammatory cytokines and promote an angiogenic and proliferative microenvironment, that probably confers a unique sensitivity of these tumours to anti-angiogenic agents. 24 patients with metastatic disease received a TKI in the first-line setting, with about half of them receiving sunitinib [13]. The toxicity profile seen was similar to those reported in previous studies amongst Asian patients, with hand foot syndrome (HFS) and diarrhoea being the predominant adverse events. Studies on sunitinib in ASPS involving Caucasian patients have shown a higher rate of HFS and neutropenia as compared to the rates reported in our study and similar Chinese studies (50% versus 20%) [6, 14]. The median PFS seen with sunitinib monotherapy (first line) was 15 months, which is in agreement with prior studies [1, 14].

Seven patients in our study cohort were exposed to immunotherapy (4-nivolumab, 3-atezolizumab). The data on the role of nivolumab in advanced ASPS continues to be disparate. The OSCAR trial studied the efficacy of single-agent nivolumab in this subset of patients, and despite the study failing to meet its specified endpoint of ORR, it did demonstrate clinically meaningful activity of nivolumab in this setting (DCR of 64%) [15]. All four patients exposed to nivolumab in our study received it in the third or subsequent line, and one patient demonstrated a sustained complete metabolic response to the agent with a response duration of 51 months. Hence, the role of nivolumab in advanced ASPS merits further investigation in larger studies. Atezolizumab (at a dosing of 840 mg 2-weekly or 1,200 mg 3-weekly) is currently the only Food and Drug Administration approved immunotherapy agent in advanced ASPS. The trial that led to this approval did not include patients with central nervous system (CNS) metastasis, and hence, the efficacy of immunotherapy in this scenario is uncertain. The best response to atezolizumab seen in our study was a partial response, which was recorded after a duration of therapy of 12 months. Delayed responses to atezolizumab have been reported in the literature, and hence, continued follow-up of these patients is required to determine ongoing responses [16].

Access to immunotherapy remains a significant therapeutic challenge in our country. The financial burden involved poses a major barrier to exposure to these agents. Other factors involved include delays in diagnosis, lack of multicentre collaboration/timely referral to specialised centres and the paucity of prospective clinical trials.

The survival of patients with brain metastasis was seen to be significantly inferior in comparison to those with non-CNS metastasis in our study, a finding that was also reported by the French Sarcoma group [17]. The outcomes in patients with brain metastasis continue to be poor irrespective of the use of anti-angiogenic agents, and hence, newer therapeutic regimens in this subset of patients are an unmet need. Two case reports from China have reported benefits in advanced ASPS with CNS metastasis with combination therapy, i.e., camrelizumab with apatinib/anlotinib [18, 19]. Hence, a combination approach might be a useful strategy in patients with CNS disease, pending more rigorous data. Camrelizumab is currently approved only in China for use in advanced ASPS.

Most studies on ASPS have included a combination of pediatric and adult patients, and data on pediatric ASPS is limited. Our series included three pediatric patients, who underwent an R2 resection and subsequently achieved a CR on adjuvant RT/sunitinib. More studies are required on pediatric ASPS as the biology of the disease may be different from adult ASPS, and responses to immunotherapy/TKI may differ. Tan *et al* [20] in their work on ASPS have shown the disease to be more indolent in the pediatric population, with a higher incidence of head and neck primaries and lower response rates to immunotherapy.

Our study has several limitations: the retrospective nature of data collection, and the absence of brain imaging in asymptomatic patients at baseline to name a few. The lack of molecular testing in all patients may have contributed to an overdiagnosis/underdiagnosis of ASPS in the study. The review of all specimens by two sarcomas pathologists was an attempt at minimalising this error. Despite these limitations, this study provides significant real-world data on the outcomes and toxicity profile of therapeutic options in ASPS in resource-limited settings.

## Conclusion

Treatment strategies in advanced ASPS have evolved from cytotoxic agents to anti-angiogenic agents to immunotherapy. Given the efficacy of both TKIs and immune checkpoint inhibitors in advanced ASPS, studies to determine the appropriate sequencing/combination of these agents are warranted. Devising an optimal treatment strategy is an unmet need in countries with limited resources, as upfront immunotherapy for all patients is not feasible. There are distinctly fewer clinical trials or prospective studies in rare cancers like ASPS. This study might act as a catalyst for further prospective studies in this rare tumour. The lack of therapeutic options in patients with brain metastasis represents a lacunae in our current understanding of this rare disease.

## Conflicts of interest

Nil.

## Funding

The study received no funding from any public or corporate organization.

## Figures and Tables

**Table 1.** Baseline characteristics of the patients.

	Number (%)
Male Female	19 (55.8%) 15 (46.2%)
Median age (Range) (years)	28 years (3–72 years)
Site of disease Extremity Trunk and viscera Head and neck	25 (73.5%) 6 (17.6%) 3 (8%)
Localised Metastatic Lungs Brain^*^ Liver Bones Non regional nodes	7 (20.6%) 27 (79.4%) 23 (85.1%) 7 (25.9%) 3 (11.1%) 5 (18.5%) 2 (5.8%)

* patients
